# Physicians' experiences and perspectives regarding the use of continuous sedation until death for cancer patients in the context of psychological and existential suffering at the end of life

**DOI:** 10.1002/pon.3450

**Published:** 2013-12-05

**Authors:** Livia Anquinet, J Rietjens, A van der Heide, Sophie Bruinsma, Rien Janssens, Luc Deliens, Julia Addington-Hall, W Henry Smithson, Jane Seymour

**Affiliations:** 1 VUB-UGent End-of-Life Care Research Group, Vrije Universiteit BrusselBrussels, Belgium; 2 Department of Public Health, Erasmus MCRotterdam, The Netherlands; 3 Department of Medical Humanities, VU Medisch CentrumAmsterdam, The Netherlands; 4 Department of Public and Occupational Health, EMGO Institute for Health and Care Research, VU University Medical CentreAmsterdam, The Netherlands; 5 Faculty of Health Sciences, University of SouthamptonSouthampton, UK; 6 University of Sheffield, Academic Unit of Primary Medical CareSheffield, UK; 7 Faculty of Medicine and Health Science, University of NottinghamNottingham, UK

## Abstract

**Objective:**

The use of continuous sedation until death for terminally ill cancer patients with unbearable and untreatable psychological and existential suffering remains controversial, and little in-depth insight exists into the circumstances in which physicians resort to it.

**Methods:**

Our study was conducted in Belgium, the Netherlands, and the UK in hospitals, PCUs/hospices, and at home. We held interviews with 35 physicians most involved in the care of cancer patients who had psychological and existential suffering and had been continuously sedated until death.

**Results:**

In the studied countries, three groups of patients were distinguished regarding the origin of their psychological and existential suffering. The first group had preexisting psychological problems before they became ill, the second developed psychological and existential suffering during their disease trajectory, and the third presented psychological symptoms that were characteristic of their disease. Before they resorted to the use of sedation, physicians reported that they had considered an array of pharmacological and psychological interventions that were ineffective or inappropriate to relieve this suffering. Necessary conditions for using sedation in this context were for most physicians the presence of refractory symptoms, a short life expectancy, and an explicit patient request for sedation.

**Conclusions:**

Physicians in our study used continuous sedation until death in the context of psychological and existential suffering after considering several pharmacological and psychological interventions. Further research and debate are needed on how and by whom this suffering at the end of life should be best treated, taking into account patients' individual preferences.

## Introduction

Continuous sedation until death (from here onwards referred to as ‘sedation’) is considered to be a last resort option to relieve unbearable and untreatable (refractory) symptoms experienced by terminally ill cancer patients 1–3. It is defined by sedation guidelines as the deliberate lowering of consciousness, continuously until the patient's death 1–3. Sedation guidelines indicate that wherever possible, the patient (and/or the patient's family) should be actively involved in decision making and give consent for the initiation of sedation. The purpose of sedation should be symptom relief and not the hastening of death 1–3.

One of the key controversies about the practice of sedation concerns its use for patients who have psychological and existential suffering at the end of life 4,5. Whereas the literature lacks uniform terms and definitions of these concepts, a common component includes nonphysical suffering such as an overwhelming sense of meaningless, hopelessness, (death) anxiety, panic, depression, sadness, anhedonia, guilt, and/or lack of any interest 6–9. Psychological and existential suffering is not uncommon among end-stage cancer patients: depression and anxiety rates range from, respectively, 3–77% and 13–79% 10. Prevalence rates of cancer patients' reported hopelessness or demoralization, and losing one's will to live or desire for death range from, respectively, 7–41% and 2–26% 11. As delirium may interfere with the recognition and control of physical and psychological symptoms, its presence should be ruled out or treated appropriately 12.

Sedation for psychological and existential suffering is considered controversial for a number of reasons. Unlike physical symptoms, it is more difficult to assess whether this suffering is truly refractory because of its subjective nature and a lack of well-established assessment tools 3,5,13. The European Association for Palliative Care recommends in its framework for the use of palliative sedation that psychological and existential distress should be confirmed by mental healthcare professionals after trials of appropriate therapy such as intermittent sedation. Only after repeated trials of intermittent sedation, continuous sedation could be considered 3. These recommendations are in line with the Belgian and Dutch sedation guidelines 1,2.

A few quantitative studies have addressed the issue of using sedation for the relief of psychological and existential suffering of severely ill patients. According to Dutch physicians, existential suffering, anxiety, and depression were amongst the indications for sedation mentioned for respectively 29%, 13%, and 30% of the patients 14. British physicians reported that in 25% of the cases in which their patient had been continuously and deeply sedated, sedation had been given because of the patient's intractable psychological suffering (whether or not in combination with other reasons) 15. In addition, a few case studies addressing this issue concluded that sedation is a useful but controversial method for treating psychological and existential suffering at the end of life but that further research is needed on the causes and therapeutic options for this suffering 13,16–18.

There is little in-depth insight in the circumstances in which physicians resort to the use of sedation for terminally ill patients with psychological and existential suffering. We performed comparative qualitative, case-based studies in three European countries with different legal, policy, and service delivery frameworks. We aimed to give insight into physicians' considerations about and descriptions of the use of sedation for cancer patients who had psychological and existential suffering at the end of life.

## Methods

This qualitative interview study is part of the larger UK – Netherlands – Belgium InternAtional SEDation (UNBIASED) study in which in-depth interviews were held with physicians, nurses, and relatives in Belgium, the Netherlands, and the UK in 2011–2012 in hospitals (oncology wards), PCUs (Belgium) or hospices (UK and the Netherlands), and at home. We refer to the published study protocol for a full description of the methods used 19.

### Study design, setting, and participants

This paper concerns the physician interviews in the three countries. Physicians were invited to take part in a face-to-face interview if they had recently taken a key role in the care of (i) a patient aged over 18 years; (ii) who had died of cancer; and (iii) who had been continuously sedated until death. We did not ask physicians how the process of continuous sedation was monitored. Senior clinical staff identified decedents who met the inclusion criteria and nominated physicians. Physicians in the three countries were interviewed about no more than four patients. If more than one physician was involved, the other involved physician was also interviewed if possible.

### Procedures

All physicians gave their informed consent to the audio taping of the interview. At the beginning of each interview, patient's and physician's sociodemographic information was obtained via the physician. Patient's anonymity was preserved. The interviews were semi-structured, supported with the use of aide-memoires that focused on physicians' recollections of the decedent's care and the use of sedation in particular and their general attitudes regarding sedation.

### Analysis

All recordings of the interviews were transcribed verbatim, and all data that could identify the physicians were removed to preserve anonymity. The Belgian and Dutch interviews were translated into English by a professional translation bureau and checked for accuracy. One researcher (L.A.) selected all physician interviews in which the physician reported on the use of sedation for patients with psychological and existential suffering. This was checked by a senior researcher (J.A.C.R.). All interviews were read by L.A. and J.A.C.R., and main themes were identified. A coding tree was designed and agreed upon on the basis of the main themes. The interviews were reread, and quotes were selected and classified under the matching main themes by L.A. and J.A.C.R. The development of the analysis was discussed with all the coauthors in telephone meetings. Qualitative analysis software (nvivo 9; QSR International Pty Ltd., 2010) was used to organize the data. Finally, quotes per main theme were selected by L.A. and J.A.C.R. for publication and were approved by all researchers.

## Results

For the present study, we explored 39 cases of patients with cancer who had psychological and existential suffering according to the physician (18 Belgium, 12 the Netherlands, and 9 UK), involving 35 physicians (13 Belgium, 11 the Netherlands, and 11 UK). Characteristics of patients and physicians can be found in Table 1. In this results section, we report on physicians' descriptions of the various types and origins of patients' suffering before sedation, the healthcare professionals and relatives involved and interventions used to manage psychological and existential suffering before sedation, and the conditions identified by physicians as necessary for the initiation of sedation.

**Table 1:** Characteristics of patients and physicians

	Patients *N* = 39	Physicians^a^ *N* = 35
Country	18	
Belgium	12	13
the Netherlands	9	11
UK		11
Care setting		
Hospital	10	7
Home	14	13
PCU/hospice	15	15
Age (years)		
<40	2	10
41–50	0	10
51–60	9	8
61–70	9	2
71–80	14	0
>80	5	0
Not stated	0	5
Gender		
Male	20	19
Female	19	16
Specialism	n/a	
Primary care		12
Palliative home care team		2
Hospital		2
Palliative support team hospital		5
Palliative care/hospice care		14
Diagnosis		n/a
Abdominal/stomach	2	
Bladder/renal	4	
Colo-rectal	6	
Brain/glioblastoma	1	
Breast	2	
Gynecological	3	
Facial maxillary/esophageal	2	
Gall bladder/pancreatic	2	
Leukemia/myelofibrosis/myeloma	2	
Lung/mesothelioma	8	
Melanoma	2	
Peritoneal	1	
Prostate	1	
Sarcoma	1	
Unknown primary	2	

n/a, not applicable.

a More than one physician could have been interviewed.

### Types of suffering

Physicians in the studied countries described a broad range of symptoms that patients experienced before they were sedated. Physical symptoms included mainly pain, dyspnea, (terminal) agitation, cachexia, or fatigue. Psychological symptoms included panic, anxiety, depression or sadness, and paranoia. Existential suffering included demoralization, despondency, sense of dependency, having difficulties being on the decline, loss of will to live, hopelessness, being ‘battle-weary’, being [mentally] exhausted, and death anxiety.

**Table 2:** Summary of main results

Continuum of suffering	From patients mainly suffering from physical symptoms to patients predominantly suffering psychologically and existentially
Three origins of psychological and existential suffering	1. Patients who already suffered from psychological problems before they became ill
	2. Patients who developed psychological and existential suffering during their disease trajectory as a reaction to their decline and approaching of death
	2a. Resigned subgroup: patients who appeared to have accepted that they were going to die soon
	2b. Resistance subgroup: patients who did not appear to have accepted that they were ill or dying
	3. Patients whose psychological symptoms were described as a direct result of their disease
Three groups of carers involved in the management of psychological and existential suffering	1. General healthcare professionals such as the physician himself/herself, other physicians, and nurses
	2. Mental healthcare professionals such as psychiatrists and psycho-oncologists
	3. Relatives
Three conditions for the use of sedation	1. Refractory (physical) symptoms
	2. The patient's short life expectancy
	3. The patient's explicit request for the use of sedation

On the basis of the physicians' accounts, we identified a continuum of patients who, at one end, suffered mainly from physical symptoms, over patients who suffered from a combination of psychological, existential, and physical symptoms, to patients who suffered predominantly psychologically and existentially (Table 2).

UK, GP, case 37: Quite a lot of her distress was psychological because she didn't want the children to see her ill [and] to know that she was ill. She developed intractable headache, intractable vomiting and some sort of gradual fixed extension of her neck that we think was metastases at the back of her neck.

Several physicians suspected that the presence of some physical symptoms was related to and may have increased the psychological and existential suffering and vice versa.

UK, palliative care physician n°2, case 32: The patient had a lot of problems with anxiety and pain which were very difficult to control all the way along. So there was felt to be quite a large psychological element to it. He was very anxious about his pain and therefore his anxiety made the pain worse and vice versa.

In patients who suffered predominantly psychologically and existentially, physical symptoms were also part of the clinical picture but were sufficiently alleviated.

Interviewer: So the pain was well controlled but the fatigue…

Belgium, Oncologist, case 19: It actually was his psychological burden… he could no longer continue.

We could not identify different patterns between the countries in perceived presence of psychological, existential, and physical symptoms.

### Origin of psychological and existential suffering

The origin of patients' psychological and existential suffering varied in the opinion of physicians. We distinguished three pathways. The first pathway concerned patients who already suffered from psychological problems such as depression, anxiety, or mourning for the death of their partner before they become (terminally) ill.

Belgium, palliative care physician, case 7: At admission, there was another symptom that we didn't know about from before. There was a pronounced problem of fear, that lady took antidepressants, so throughout her life she had been depressed.

Patients to whom the second pathway applied developed psychological and existential suffering during their disease trajectory as a reaction to their decline and approaching of death. In this second group, there were two subgroups with regard to the patients' way of coping with their approaching end. Patients of the ‘resigned’ subgroup usually appeared to have accepted that they were going to die soon and were often described to have had ‘enough of it’.

Belgium, oncologist, case 5: The patient asked the question and took the decision. ‘I want to sleep, I do not want to wake up anymore’. He was just totally bedridden, he couldn't do anything anymore, he had no quality of life and he also had a very bad life expectancy. He just had enough.

The ‘resistance’ subgroup did not appear to have accepted that they were ill or dying and were described as angry, anxious, and in panic, not wanting to be dependent on others and finding it hard to accept that their health was declining.

Netherlands, palliative care physician, case 29: The pain was well controlled, but the man had a fear of going to bed, because if he went to bed he could die. He was very afraid of that. He was really fighting it.

The third pathway concerned patients whose psychological symptoms were described as a direct result of their disease. Examples are patients who suffered from a neurological deficit because of a brain tumor, with aggressive behavior, agitation, or confusion as a consequence. They were often not (fully) competent anymore according to the physicians.

Belgium, palliative care physician, case 3: Sedation was used for obvious symptoms of fear, paranoia, suspicion, aggression and a very difficult communication in the context of a central tumor with severe communication disorders.

### Management of psychological and existential suffering

Physicians in the three countries described how they had managed the patient's psychological and existential suffering before using sedation. They described three groups of carers that were involved and who had used several psychological as well as pharmacological interventions.

The first group of carers consisted of general healthcare professionals such as the physician himself/herself, other physicians, and nurses. A pharmacological intervention described was the administration of medication such as anxiolytics, antidepressants, or benzodiazepines (in smaller dosages).

UK, GP n°1, case 38: We give people diazepam in exactly the same way that we would use diazepam for acute anxiety and psychological distress in people who weren't terminally ill.

Sometimes, intermittent sedation had been used by physicians as a ‘time-out’.

UK, palliative care physician, case 34: Talking and communicating is one side of it but you can't be there all the time and the patient just wants some time out. We sometimes talk about it in those terms with the patient: ‘When things are getting really on top of you, an injection that will put you to sleep, might just help you get over that really bad period. That will give you a sleep for four, six hours, but then when you wake up we can see how things are then.’ And patients and doctors often refer to that as giving somebody Midazolam [a sedative] for time out.

Not all physicians had used medication to alleviate the suffering. One of them explained that drug treatment for psychological symptoms needs several weeks to take effect, while the patient only had days to live. The physician added that drug treatment of psychological symptoms in terminally ill patients may meet additional effectiveness problems, for example, when the liver fails to metabolize medications.

Netherlands, oncologist, case 21: The problem was that a drug treatment for depression needs at least several weeks to take effect. And because her liver was not working, you cannot give her medication. So her depression was not treatable with medication.

Sometimes, psychological interventions such as talking and spending extra time with the patient had been applied.

UK, palliative care physician n°3, case 31: He was a gentleman who the team probably spent more time than average with, because he was anxious we tried using more conservative measures. Sometimes just talking things through with patients can be quite effective.

Some other physicians stated that nurses tended to be more involved in the psychological management than they themselves.

UK, palliative care physician, case 39: The nurses tend to do maybe a bit more of the psychological and social issues. Although, clearly there's a big overlap and we all do a bit of everything. But that's the way we tend to divide it up.

Furthermore, psychological interventions were not by all physicians deemed suitable for all patients, for instance, because the patient needs to have sufficient energy, should be competent, must not be too exhausted and should want to get involved.

Belgium, palliative care physician, case 18: As a doctor you can reasonably estimate what can still be done and what can't. And to what extent the patient still wants, and still brings up the energy to get started with something. Because for a psychologist, or a consultant, the patient must indeed certainly still have a clear capacity of thinking and not be too tired.

The second group of carers who was involved in the management of the patient's psychological and existential suffering included mental healthcare professionals. These concerned psychiatrists, psychologists, psycho-oncologists, counselors, and pastors. Their main involvement concerned, according to the respondents, talking to the patient (or the patient's family).

UK, palliative care physician, case 31: The other people we involved were the psychiatrist, because he'd had depression and he'd been depressed for some time before he became ill. He'd also seen the psycho-oncologist when he came in, and [she/he] just talked to him.

Sometimes, a psychiatrist had been called in by the physician to determine the refractory nature of a psychological symptom.

Netherlands, oncologist, case 21: In extensive consultations with the psychiatrists we determined that the depression was refractory because we had no options to treat it.

However, not all physicians reported on the involvement of a specialist, and some said explicitly that they had not done so.

Belgium, palliative care physician, case 18: The moral counsellor, that's something we seldom use, this is a Catholic hospital. They're there, but they [psychologist and moral counsellor] are not involved that much, unless the patient really wants it.

The third group was the patient's relatives. They were described as talking to and supporting the patient.

Belgium, GP, case 10: I had the impression that we were lucky that the informal care provided by the family was very good, that it made the patient very calm.

Similar types and treatments of psychological and existential suffering and groups of carers involved in these treatments were reported by physicians in all three countries.

### Conditions for the use of sedation

Several physicians described that for them, a condition for the initiation of sedation for psychological and existential suffering was the presence of refractory symptoms.

Netherlands, oncologist, case 21: Frankly, we were a bit happy that the lady had refractory symptoms. Initially we said sedation is not possible, because we do not have refractory symptoms.

Moreover, the presence of *physical* refractory symptoms was an important condition to perform sedation for most, but not all, physicians.

Netherlands, oncologist, case 21: Terminal restlessness, delirium, dyspnoea and pain. These are the most common reasons for someone to sedate. I had never experienced using sedation for refractory depression, and I think in our unit it is also unprecedented.

Some said that they would be reluctant to sedate for mainly psychological and existential suffering; and some said that in the case of psychological symptoms, they would wait until some refractory physical symptoms arise.

Interviewer: You said that those physical problems must also increase before you start with the sedation?

Belgium, palliative care physician, case 18: Yes. Purely based on psychological symptoms is a lot harder, the physical deterioration must most certainly be there as well.

Yet, the refractory nature of symptoms was not for all physicians a necessary condition for the use of sedation.

Netherlands, palliative care physician, case 20: I just couldn't put my finger on what it was. It was behavior that was not possible to correct, in which she lingered, where she was frightened. I'm not sure whether it was a delirium, or behavioral disorders that can occur from the stroke. My colleague said: you're really with your back up against a wall here. You do not really have a refractory symptom, but by God you have no idea what else you could possibly do about this. Do you have to interpret the guidelines so broadly? That sometimes makes it difficult.

Similar results were found in each of the studied countries.

Another condition that was frequently described in the three countries was a patient's short life expectancy.

Interviewer: What are the criteria [for the use of sedation] you uphold?

Netherlands, palliative care physician, case 19: First that's a life expectancy of less than two weeks. I usually keep to a week, because death needs to be close by.

This condition was more present in the accounts of Dutch and British physicians compared with what Belgian physicians reported.

A last condition according to several physicians was patient's explicit request for the use of sedation or request to be asleep and not wake up anymore.

Belgium, palliative care physician, case 18: So [among the indications for sedation are] a [short] prognosis, and a second point of course is that the patient asks for it [sedation].

## Discussion

This study provides insight into physicians' experiences with continuous sedation until death in the context of psychological and existential suffering, physicians' reported management of this suffering, and conditions identified by physicians as necessary before resorting to sedation at the end of life.

Our study benefited from the use of in-depth face-to-face interviews, allowing us to explore physicians' experiences and perspectives on a highly debated subject. However, our results cannot be generalized in a statistical way to the whole population of physicians or to physician specialty in particular because of the relatively small number of cases and interviews. Instead, our findings may provide insights that may be transferrable to similar clinical cases and contexts. Additionally, our data gathered by interviews are dependent on the subjective experience and interpretation of both the physician and the interviewer. Although our retrospective design does not preclude recall bias, this is limited in most of the cases by minimizing the time between the patient's death and the interview with the physicians to 2 months. In a few cases, this was longer than 2 months, and the risk of recall bias could have been larger in these cases.

Physicians in the three countries had used sedation for a continuum of patients who were mainly suffering from physical symptoms to patients predominantly suffering psychologically and existentially. The patients' psychological and existential suffering could have been present before the patient became (terminally) ill or developed and worsened during the patient's disease trajectory. As stated by the respondents and confirmed by literature, the existence of (unrelieved) physical symptoms may have led to the psychological and existential suffering and vice versa 8.

In the accounts of the physicians, the various manners in which they had attempted to relieve the patient's psychological and existential suffering and the considerable efforts that this often took were central. In several of our case studies, in addition to being near the patient and building a trusting relationship, physicians had involved mental healthcare professionals, and they often had administered medication or intermittent sedation to relieve suffering before using continuous sedation. Similar interventions were reported by physicians in a Japanese study in PCUs for terminally ill cancer patients suffering from refractory psycho-existential issues before sedation 7. In the literature, a number of psychotherapeutic interventions have been proposed, such as cognitive–behavioral therapy, group therapy, existential therapy, and psycho-education, but research does not seem to support the value of one approach over others in cancer patients 9,20.

Nevertheless, our study shows that pharmacological and psychological interventions may not be effective or appropriate for all patients. Physicians reported that pharmacological therapies other than sedation were considered but were precluded by the patient's short life expectancy or liver failure. It is supported by the literature that drug therapy takes time to have effect and that drug metabolism may be altered and decreased because of an organ failure in terminally ill patients 21,22. Recently however, there has been renewed interest, especially in the United States, in the use of psychostimulants for treating depression in physically ill patients. Although the evidence base is still limited, its use has been recommended because of its potential to enhance mood, energy, and arousal within hours 23. Also, antipsychotics may help reduce delirium symptoms as well as psychological symptoms 24. Intravenous Ketamine, an analgesic, has also been shown to contribute to the rapid treatment of depressive symptoms in palliative patients 25.

A main condition for the initiation of sedation for psychological and existential suffering was for most physicians the presence of refractory symptoms. This conforms with the sedation guideline recommendations 1–3. There was often reluctance among physicians in the studied countries to sedate for mainly psychological and existential suffering. Another study also found physicians to be less in favor of or opposed to using sedation to unconsciousness for existential suffering 26. Physicians in our study explained that they were reluctant to use sedation in these cases for personal reasons and that they would wait for the appearance of coexisting refractory physical symptoms. It is also possible that physicians are reluctant to use sedation for psychological and existential suffering because this suffering may fluctuate as death approaches and is usually amenable to pharmacological and psychological treatments such as the dignity model intervention designed for patients at the end of life 27,28. This reluctance may heighten the risk of not exploiting enough the potential for interventions. Further, for the physicians in our study, the patient's short life expectancy and an explicit request to be asleep and not to wake up again were deemed as important. No differences were found between the studied countries, except that the patient's short life expectancy as a condition for the use of sedation seemed less important for Belgian physicians. This does not conform with sedation guideline recommendations 1–3. Possibly, these physicians primarily focused on adequate symptom relief regardless of the patient's short life expectancy.

In conclusion, physicians in our study resorted to the use of continuous sedation until death for cancer patients with psychological and existential symptoms after considering several pharmacological and psychological interventions and involving healthcare professionals. Extensive debate and research on when, how, and by whom psychological and existential suffering at the end of life should be best treated are urgently needed, taking into account the individual differences between patients regarding their preferences and wishes and healthcare status 9. Studies should focus on how mental healthcare can be most effectively incorporated into routine medical care and especially palliative care, taking into account patients' individual preferences 29,30. As several studies have shown that existential distress such as demoralization and hopelessness is linked to terminally ill patients' desire for death, ethical debate should focus on whether and under which circumstances a far reaching medical practice such as continuous sedation until death is a desirable response to psychological and existential suffering at the end of life 31,32.

## Contributors

The research on which this paper is based is linked to a larger project, the ‘UNBIASED’ study, which is a collaboration between research teams in the UK, Belgium, and the Netherlands. All authors and members of the UNBIASED consortium were involved in the study concept, design, and interpretation of data. The data were collected and subject to preliminary analysis by JS, JB, LA, KR, SB, and JR. All authors had full access to all of the data in the study and can take responsibility for the integrity of the data and the accuracy of the data analysis. The first drafts of the paper were written by LA and JR, with redrafting by other coauthors. All authors have seen and approved the final version. The lead author, LA, is the guarantor.

The following are members of the UNBIASED consortium: Julia Addington-Hall (University of Southampton, Southampton, UK), Livia Anquinet (Vrije Universiteit Brussel, Brussels, Belgium), Jayne Brown (De Montfort University, Leicester, UK), Sophie Bruinsma (Erasmus MC, Rotterdam, the Netherlands), Luc Deliens (Vrije Universiteit Brussel, Brussels, Belgium and VU University Medical Centre, Amsterdam, the Netherlands), Nigel Mathers (University of Sheffield, Sheffield, UK), Sheila Payne (Lancaster University, Lancaster, UK), Kasper Raus (Ghent University, Ghent, Belgium), Judith Rietjens (Erasmus MC, Rotterdam, the Netherlands and Vrije Universiteit Brussel, Brussels, Belgium), Clive Seale (Brunel University, Uxbridge, UK), Jane Seymour (University of Nottingham, Nottingham; UK), W. Henry Smithson (University of Sheffield, Sheffield, UK), Sigrid Sterckx (Ghent University, Ghent, Belgium), Rien Janssens (VU University Medical Centre, Amsterdam, the Netherlands), and Agnes van der Heide (Erasmus MC, Rotterdam, the Netherlands).

## Ethics committee

The study was approved in the UK by the Leicestershire, Northampton, and Rutland Research Ethics Committee 1, reference number 10/H0406/57; in Belgium by the Ghent University Hospital Ethics Committee, reference number B670201010174; and in the Netherlands by the Erasmus MC Medical Ethical Research Committee, reference number NL33327.078.10, V03. Each participant gave written informed consent before taking part.
